# Effect of fragrance use on discrimination of individual body odor

**DOI:** 10.3389/fpsyg.2015.01115

**Published:** 2015-08-07

**Authors:** Caroline Allen, Jan Havlíček, S. Craig Roberts

**Affiliations:** ^1^Division of Psychology, School of Natural Sciences, University of Stirling, Stirling, UK; ^2^Department of Zoology, Charles University, Prague, Czech Republic

**Keywords:** deodorant, olfaction, body odor, identification, triangle test, smell

## Abstract

Previous research suggests that artificial fragrances may be chosen to complement or enhance an individual’s body odor, rather than simply masking it, and that this may create an odor blend with an emergent quality that is perceptually distinguishable from body odor or fragrance alone. From this, it can be predicted that a new emergent odor might be more easily identified than an individual’s body odor in isolation. We used a triangle test paradigm to assess whether fragrance affects people’s ability to distinguish between individual odors. Six male and six female donors provided axillary odor samples in three conditions (without fragrance, wearing their own fragrance, and wearing an assigned fragrance). In total, 296 female and 131 male participants selected the odd one from three odor samples (two from one donor, one from another; both of the same sex). We found that participants could discriminate between the odors at above chance levels in all three odor conditions. Olfactory identification ability (measured using Sniffin’ Sticks) positively predicted discrimination performance, and sex differences in performance were also observed, with female raters being correct more often than men. Success rates were also higher for odors of male donors. Additionally, while performance was above chance in all conditions, individual odor discrimination varied across the three conditions. Discrimination rate was significantly higher in the “no fragrance” condition than either of the fragranced conditions. Importantly, however, discrimination rate was also significantly higher in the “own fragrance” condition than the “assigned fragrance” condition, suggesting that naturally occurring variance in body odor is more preserved when blended with fragrances that people choose for themselves, compared with other fragrances. Our data are consistent with the idea that fragrance choices are influenced by fragrance interactions with an individual’s own body odor.

## Introduction

There is a wealth of evidence supporting the availability of various cues from human body odor. These cues concern a wide range of variables from emotion (Chen and Haviland-Jones, 2000; Fialová and Havlíček, 2012), menstrual cycle stage (Singh and Bronstad, 2001; Havlíček et al., 2006) through to health status (Moshkin et al., 2012). The aforementioned cues represent transitory changes in the perceptual qualities of body odor, and, despite these changes, individuals seem to maintain an underlying idiosyncratic quality to their odor which can be readily distinguished by others. Research has found that relatives can reliably discern the odor of a sibling from that of a stranger of the same age and sex (Porter et al., 1986), individuals can pick out a shirt worn by themselves out of 100 worn by others (Lord and Kasprzak, 1989), and the odors of identical twins can be matched at above chance levels by human sniffers, even when the siblings are living apart (Roberts et al., 2005). These findings are further supported by research showing that humans have distinct and reproducible “fingerprints” comprised of specific volatile compounds in their body odor (Penn et al., 2007). Human body odors have also been found to contain cues to genetic similarity at the major histocompatibility complex (MHC), with research finding individuals to be capable of discriminating between MHC types, which may lead to adaptive mate choice for heterozygous offspring (Wedekind et al., 1995; Havlíček and Roberts, 2009).

There are a multitude of benefits incurred by an individual who can discriminate between conspecifics using olfactory information. For example it has been suggested that in the mother-infant relationship, odor recognition and detection are important for both the forming of an attachment, and for inducing feeding (Raimbault et al., 2007). It has been found that mothers can discriminate the smell of their own offspring from others (Porter et al., 1983; Ferdenzi et al., 2010), with neonates also reportedly being capable of discriminating between their own mother’s axillary odors and that of an unfamiliar lactating female (Cernoch and Porter, 1985). Odor also appears to be important for human mate choice. Facial and body symmetry have been posited as reflecting an individuals’ developmental stability; a potential indicator of genetic quality. This is therefore a potentially useful mate-choice relevant cue that varies across individuals. Studies have found that those who have higher levels of facial and body symmetry are rated as looking and smelling more attractive (Rikowski and Grammer, 1999; Thornhill and Gangestad, 1999).

Although these findings suggest that body odor discrimination is important, personal odor is often “modified” with the use of artificial fragrances (Roberts and Havlíček, 2012), with the conscious evaluation of body odor having a long history of negative connotations within numerous cultures (Schleidt et al., 1981). Reduction of ones’ ability to detect individual characteristics of body odor would, at first sight, appear to be problematic given the information that can be gained from an individuals’ odor and its influence in various social interactions. However recent research suggests that, rather than masking odor entirely, fragrances may in fact be chosen to complement and perhaps enhance the volatiles present in an individuals’ body odor. For example, Milinski and Wedekind (2001) found that MHC genotype correlated significantly with an individuals’ “liking” of a fragrance compound, which they argue suggests that humans choose fragrances to amplify genetic cues present in their odor. In keeping with this, Lenochová et al. (2012) found that mixtures of participants’ body odor with their perfume of choice were perceived by female raters to be more pleasant than a mixture containing a randomly assigned perfume, even when controlling for the pleasantness of fragrances. This suggests that fragrances are chosen to work in tandem with individual body odor, potentially enhancing an individuals’ personal olfactory fingerprint.

In light of this, the current study aimed to investigate the effect of fragrance use on the perceived individual quality of body odor, thus further investigating whether fragrances may mask or enhance idiosyncratic cues in body odor. To do this, odor samples were collected from individuals who were matched on deodorant brand use. In order to assess participants’ ability to discriminate between these odors, triangle tests were conducted in which participants had to select the “odd one out” from three odors in which two were from the same individual. This test was conducted with both unfragranced body odor samples and, from the same individuals, blended samples of body odor and fragrance where the fragrance was the donor’s usual brand of choice. The former allowed us to assess underlying ability for discrimination of body odors, while the latter allowed us to assess the impact of fragrance on idiosyncratic information available in that body odor. Finally, the test was repeated using samples containing body odor and a fragrance that was assigned to the donor by the experimenters (following Lenochová et al., 2012). This enabled us to investigate whether fragrance is specifically chosen by an individual in order to enhance their idiosyncratic biological information.

Based on previous findings showing that humans are capable of discriminating between individual odors, we expected that, at least in the unfragranced body odor condition, participants would be able to identify the odd one out at an above chance level. Similarly, in view of the findings of Lenochová et al. (2012), we predicted that performance would be at above chance levels for assessments of body odor and donors’ own deodorant blends. Indeed, if body odor and fragrance do combine to form a new emergent odor, task performance might even exceed that of the no fragrance condition. In contrast, we hypothesized that participants would perform worse in the condition employing samples containing an assigned deodorant, as this fragrance had not been chosen by the donor and so might clash with the idiosyncratic body odor.

## Materials and Methods

The study received ethical approval from the University of Stirling Psychology Ethics Committee.

### Odor Collection

All donors provided informed consent. Odor samples were collected from six men (mean age ± SD = 24.5 ± 5.24, range 19–32) and six women (mean age ± SD = 21.17 ± 2.93, range 18–26), all of whom reported being heterosexual, non-smokers who regularly wore deodorant. As cyclical hormonal changes related to the menstrual cycle can affect the perceptual quality of body odors (Kuukasjärvi et al., 2004; Havlíček et al., 2006) we recruited only female donors who reported using hormonal contraception. Donors were additionally selected based on their current deodorant use, with all males reporting using the same commercially available fragrance (Lynx Africa—deodorant body spray). Female donors did not all use the same deodorant, but were selected so that there were two individuals each using the same deodorant (two using Sure Crystal Invisible, two using Nivea Pearl and Beauty and two using Dove Go Fresh Pomegranate and Lemon—all antiperspirant deodorants). This ensured that, for both men and women, triangle tests could be established utilizing donor pairs who used the same fragrance. All six female donors reported shaving their armpits during the study, whereas all male donors reported not shaving their armpits.

Each donor provided three axillary odor samples; one whilst wearing no deodorant (no fragrance), the second whilst wearing their own deodorant (own fragrance) and the third whilst wearing a deodorant provided by the experimenter (assigned fragrance). The assigned deodorant was chosen on the basis that it was not currently, or previously, used by any of the donors, with the six males receiving the same commercially available product which was designed for men (Adidas Ice Dive—a deodorant body spray), and the six female donors receiving the same commercially available deodorant which was designed for female use (Vaseline Active Fresh—an antiperspirant deodorant).

Odor collection took place on three consecutive days, with donors being instructed to shower before and between each session using fragrance free soap (Simple Pure^™^) which we provided. Donors were instructed to only use the soap provided, and the deodorants (only on the relevant days), and to avoid all other fragranced products. After showering, participants attached cotton pads to their armpits using surgical micropore tape. On the second and third days, after showering, participants were instructed to apply deodorant to both armpits (own deodorant on the second day, assigned deodorant on the third day), in their usual way, before attaching the cotton pads. These were left in place for 24 h, after which they were removed, placed in sealed plastic bags, and returned to the experimenter (within 2 h) where they were frozen at –30°C until use. Samples were removed from the freezer 2 h prior to test use, so that they could thaw, and placed back in the freezer at the end of each test session. Previous studies suggest that freezing and thawing of samples has little impact on perceptual qualities of odors (Roberts et al., 2008; Lenochová et al., 2009). In order to reduce the effect of any extraneous odors on the samples, and in line with previous research, participants were instructed to avoid being in smoky places, drinking alcohol, exercising, eating particularly strong smelling foods (e.g., curry, garlic), having sex and sharing a bed with another person starting from the day prior to odor collection and also during odor collection (Kohoutová et al., 2011; Lenochová et al., 2012).

### Triangle Test Participants

All participants were visitors at the Centre for Life in Newcastle upon Tyne. The tests for male and female odor samples were completed by independent sets of participants. In total, 238 participants (65 men; mean age ± SD = 40.15 ± 16.15, range 16–76 and 173 women; mean age ± SD = 41.97 ± 13.36, range 17–79) completed the test with male odor samples. A set of 189 participants (66 men; mean age ± SD = 41.11 ± 14.75, range 16–76 and 123 women; mean age ± SD = 38.06 ± 14.83, range 16–78) completed the test with female odor samples.

### Triangle Test Procedure

Participants provided informed consent and basic demographic information (age and sex). The nature of the task was explained in advance, and participants were told that they would be smelling samples of body odor and fragrance. Each participant was then presented with three 500 ml clear glass conical flasks, with aluminum foil caps, containing odor samples. Two of these odor samples were from the same individual, and the third was from a different donor of the same sex. For the donor who only presented one sample, the right axillary sample was used. Participants were informed that one of these was different from the rest, and they were instructed to remove the tinfoil covering and smell each flask before identifying the odd one out.

Within each triangle test donor samples were paired so that each pair used the same deodorant (males paired with males and females paired with females). There were three odor conditions, with each triangle test having all three samples containing either no fragrance, own fragrance or assigned fragrance and participants were blind to these. Each participant took part in one session during which they completed one triangle test in each of the three odor conditions, with each test involving a different donor pair. Each session used either all male or all female donor samples, and consequently each participant was exposed to either all of the female or all of the male samples (see Table 1). After sample use each glass flask was cleaned using a fragrance free detergent (Neutracon, Decon Laboratories Ltd.) and allowed to dry prior to the next test session. Both male and female samples were used in three separate test sessions (Table 1) each of which was conducted over approximately a day and a half. This meant that samples were thawed and used for 5–6 h before being refrozen and thawed the next day where they were used for a further 2–4 h (depending on the number of visitors at the center). Samples were treated in the same way (i.e., time of use) across the three conditions. Table 1 shows the number of participants who took part in each test session.

**TABLE 1:** **Donor pairings used in each triangle test**.

**Test session**		**Donors used in each condition**		
		**No fragrance**	**Own fragrance**	**Assigned fragrance**
**Male donor samples**				
A	*n* = 68 mean age ± SD = 42.69 ± 13.45	1 and 2	3 and 4	5 and 6
B	*n* = 74 mean age ± SD = 41.62 ± 13.80	3 and 4	5 and 4	1 and 2
C	*n* = 96 mean age ± SD = 40.49 ± 14.97	5 and 6	1 and 2	3 and 4
**Female donor samples**				
D	*n* = 59 mean age ± SD = 42.76 ± 15.47	7 and 8	11 and 12	9 and 10
E	*n* = 71 mean age ± SD = 36.11 ± 14.43	9 and 10	7 and 8	11 and 12
F	*n* = 59 mean age ± SD = 39.10 ± 14.06	11 and 12	9 and 10	7 and 8

Each participant took part in one session, and were therefore exposed to all three conditions, with three odors in each (two of the same, one of a different donor), all of which were of the same sex. Consequently each participant was exposed to either all of the male donor samples OR all of the female donor samples. Mean participant age ± SD is shown for each test session.

Additionally, each participant completed the Sniffin’ Sticks Screening test. This is a 12-item cued odor identification test (Hummel et al., 2007a) which assess ability to verbally label common odors. It employs the use of odor dispensing devices, shaped like pens. Participants sniff each of these and then must select the correct label for the odor from a choice of four words. The resulting score is the sum of correct answers. This was completed after the triangle test.

## Results

**FIGURE 1:**
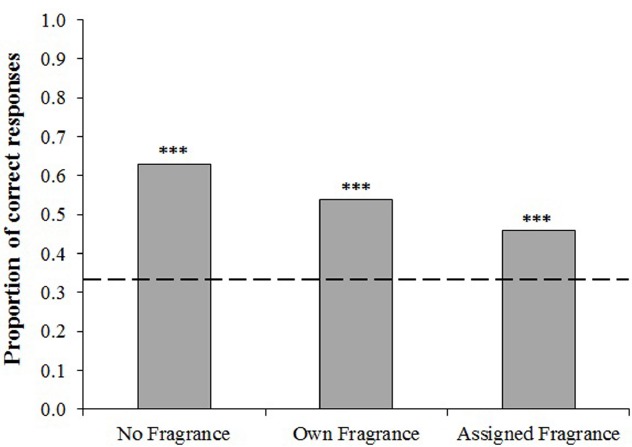
**Proportion of participants who correctly chose the odd one out on the triangle test.** Dashed line indicates the proportion of correct responses which would be expected by chance (0.33). Binomial tests indicate significance above chance level ***p < 0.001.

Binomial tests were first conducted to compare the observed frequency of correct scores against that expected by chance (in this case 0.33). For each condition, participants were able to discriminate between the odors at a level significantly above chance (all *p*’s < 0.001, Figure 1). A Chi-squared test indicated that there was a significant difference between the number of correct responses achieved in the three odor conditions, *χ*^2^(2) = 23.87, *p* < 0.001.

**FIGURE 2:**
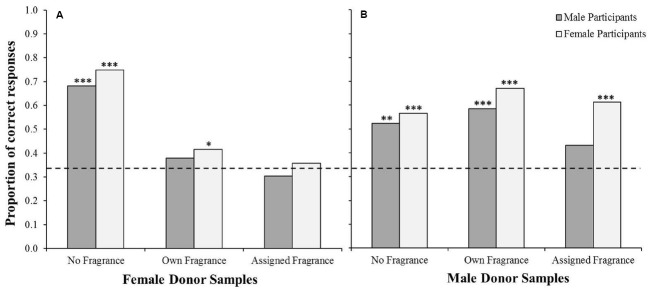
**Proportion of male and female participants who correctly chose the odd one out on the triangle test when using female samples (A) and male samples (B).** Dashed line indicates the proportion of correct responses which would be expected by chance (0.33). Binomial tests indicated significance above chance *p < 0.05, **p < 0.01, ***p < 0.001.

In order to investigate these differences further, a binary logistic regression was conducted. The dependent variable was the participants’ response in each test (correct, incorrect) and we included five candidate predictor variables in the model; donor sex, participant sex, participants’ scores on the Sniffin’ sticks test, participants’ age, and odor condition (“no fragrance,” “own fragrance,” “assigned fragrance”). Performance on the Sniffin’ sticks test significantly and positively predicted participants’ performance on the triangle tests, Exp (B) = 1.175, *p* < 0.001, as did participant sex, Exp (B) = 0.777, *p* = 0.048 (females having a higher proportion of correct responses, 0.57, compared to males, 0.48). The effect of donor sex was also significant, *p* = 0.001, Exp (B) = 1.503, such that there was a higher proportion of correct responses when assessing male samples (0.59) compared with female samples (0.49). Importantly, odor condition was found to be a significant predictor of test performance, *p* < 0.001. Orthogonal planned contrasts revealed that the proportion of correct responses was higher in the “no fragrance” condition than that of the two fragranced conditions, Exp (B) = 1.749, *p* < 0.001, and higher in the “own fragrance” condition than that of the “assigned fragrance” condition, Exp (B) = 1.375, *p* = 0.03. The model also revealed a significant interaction between odor condition and donor sex, *p* < 0.001, with participants returning more correct responses when assessing female samples in the “no fragrance” condition, Exp (B) = 0.175, *p* < 0.001, while the proportion of correct responses was higher in male samples in the “own fragrance” and “assigned fragrance” conditions, Exp (B) = 1.094, *p* = 0.757 (Figure 2). There was no significant interaction between participant sex and performance across the three conditions, *p* = 0.603. Interestingly, while it is well documented that olfactory ability declines with age (Hummel et al., 2007b) there was found to be no effect of participants’ age on task performance, Exp (B) = 0.998, *p* = 0.674. We did, however, find that participants’ age was significantly negatively correlated with performance on the olfactory identification test, *r* = –0.207, *n* = 420, *p* < 0.001, with older individuals performing worse than younger individuals.

Finally, in order to further investigate the significant interaction between odor condition and donor sex, we repeated the analysis separately for responses to male and female samples by male and female participants (Figure 2). Binomial tests indicated that, for female odor samples, men correctly discriminated the odors at proportions above chance in the no fragrance condition, *p* < 0.001 (0.68 correct), but not the own fragrance (0.38 correct) or assigned fragrance condition (0.30 correct), whereas women were correct at an above chance level in both the no fragrance, *p* < 0.001 (0.75 correct), and the own fragrance conditions, *p* = 0.03 (0.41 correct), but not the assigned fragranced condition (0.36 correct, see Figure 2A). However, performance was higher for male odor samples, with men performing at a significantly above chance level in both the no fragrance, *p* = 0.001 (0.52 correct), and the own fragrance conditions, *p* < 0.001 (0.58 correct), but not in the assigned fragrance condition (0.43 correct), and women performing above chance in all three conditions, no fragrance *p* < 0.001 (0.57 correct), own fragrance *p* < 0.001 (0.67 correct), and assigned fragrance *p* < 0.001 (0.61 correct; see Figure 2B).

## Discussion

Our study aimed to investigate the impact of artificial fragrances on the perception of individual body odors, and in turn, to investigate whether fragrances might either mask or enhance idiosyncratic information available in odors. This was achieved using a triangle test paradigm, with participants identifying the “odd one out” from three odors, either with no fragrance, the donors’ own fragrance, or an experimenter assigned fragrance. As expected, the discrimination rate was highest in the “no fragrance” condition, followed by the “own fragrance” and then the “assigned fragrance” conditions. Furthermore, participants’ performance on the triangle test was mediated by their olfactory ability, as assessed using the Sniffin’ Sticks identification task. Individuals with higher identification scores performed better in the triangle tests. We found no relationship between participants’ age and their performance on the task, which might at first sight be surprising given that olfactory ability tends to decline with age (Hummel et al., 2007b). However, this is likely explained by the inclusion of scores from the Sniffin’ Sticks task in the model. As would be predicted, these scores were negatively correlated with participants’ age.

Our results also indicate that female participants performed better on the triangle tests than male participants did. This is perhaps unsurprising as it has repeatedly been reported that women tend to outperform men on various aspects of olfactory perception (Brand and Millot, 2001; Cardesín et al., 2006; Doty and Cameron, 2010). Additionally, previous work has also found women to outperform men in specific tasks of body odor identification (Schleidt, 1980) and self-recognition of body odors (Platek et al., 2001).

Irrespective of the participant sex differences reported, all participants were good at discriminating between odors, performing at a significantly above chance level in the no fragrance condition, supporting previous findings such as those of Lord and Kasprzak (1989). Furthermore, participants’ performance was also at a significantly above chance level in both of the deodorant conditions, lending further support to the idea that fragrance does not mask information present in body odor. More importantly however, was the finding that performance was significantly better in the “own fragrance” condition compared to the “assigned fragrance” condition. This indicates that fragrance-body odor blends involving individually preferred fragrances are qualitatively different from blends involving randomly selected fragrances. Such findings further substantiate claims by Milinski and Wedekind (2001) and Lenochová et al. (2012) that fragrances may, perhaps unintentionally, be chosen to complement body odors. However, it does appear that, while participants’ performance when assessing blends with the fragrance of choice was better than with assigned fragrances, it was poorer than when assessing body odor alone. This suggests that the emergent quality of the blend does not appear to actively enhance individuality, even though it does not appear to mask it either.

It must be noted, however, that the current study raised some interesting questions regarding differences in discrimination between odors when using male and female samples. For female odors the findings were largely consistent with the overall analysis, such that unfragranced samples were the easiest to discriminate, followed by own fragranced samples and then assigned fragranced samples, and with discrimination of assigned fragrance samples being at about chance levels (though performance in the two fragranced conditions was not significantly different). However, this pattern was not evident in male samples with participants performing in all conditions at a significantly above chance level, and with there being no significant difference between participants’ performance across the three conditions.

It is possible that this finding was driven by the quality of the male odors. Male odors appear to be more intense and distinctive than female odors, and it may therefore be easier to discriminate between them even in the presence of a fragrance. In support of this, previous studies have suggested that discrimination between male and female odors is probabilistic, with sex classifications being related to the perceived intensity of the odors: stronger, more intense odors are more likely to be judged as male than weaker ones, regardless of the actual sex of the odor donor (Doty et al., 1978; Doty, 1981). An alternative, or contributory explanation is that the male fragrances used here were all deodorants, containing only fragrance and compounds which reduce the presence of odor causing bacteria, whereas the female fragrances used were all antiperspirant deodorants, and thus additionally contained compounds which inhibit the production of sweat. This may have also contributed to different levels of intensity in the male and female samples, but intensity was not assessed by our raters and we therefore cannot confirm this. One further possible explanation is that the assigned fragrance for the male donors was in some way perceptually different than that given to the female donors, making discrimination of male odors easier. Either of these suggestions, in isolation or taken together, may provide an explanation for the improved performance with male samples, and future research should aim to investigate this further by including intensity ratings of the individual odors, with and without fragrances, as well as ratings of fragrance intensity in the absence of body odor, or perhaps by utilizing a unisex fragrance for the assigned condition.

Furthermore, due to the setting in which the experiment took place we were somewhat restricted as participants did not have time to complete more than three tests (taking approximately 10–15 min per participant). Conducting the study in this environment presented a trade-off between the number of participants completing the test and the number of tests they each completed, which allowed us to obtain a very good sample size with a large and representative age range. Importantly the odor conditions were balanced, with each participant completing a test in each odor condition, which is the critical element of the experimental design. It should also be noted that while we recruited a large sample of participants, there were only six donors of each sex, and future research should employ a larger number of donors in order to present a more representative range of odors.

Despite this, the current study benefits from adopting a more ecologically valid methodology than has previously been used. Previous research investigating the effects of fragrances on body odor tend to use perfumes as opposed to deodorants (Havlíček and Roberts, 2013). There is a good reason for this; perfumes are solely fragrance, whereas deodorants combine fragrance and odor suppressants. However, deodorants are widely used, with one study reporting that between 82.7 and 93.3% of 17,000 individuals sampled in the UK indicating they used a deodorant either daily or on most days (Rodriguez et al., 2013). Thus, assessment of the effects of deodorants, as well as perfumes, are important to understand the cultural effects of modern patterns of fragranced products on odor perception. It is also noteworthy that individual discrimination was possible despite the odor-suppressing qualities of deodorants and their anti-microbial action, and that because of this the current findings may actually underestimate discrimination rates. Furthermore, it was in the odor samples provided by women, who used antiperspirant deodorants, that identification was improved with the use of a chosen versus an allocated fragrance, lending additional support to the importance of fragrance/body odor blends in identification, rather than a reduction of sweat or body odor.

The findings from this study help to reveal just how complex the perception and holistic affective response to fragrance users by other individuals around us is in real-life interactions. As mentioned above, the majority of people wear some form of fragrance on a daily basis (Rodriguez et al., 2013). It is also likely to be the case that when entering a mate choice arena, for example when going on a date or for a night out in a nightclub, that an even larger proportion of individuals will be wearing fragranced products. Given this, it is most likely that encounters with new individuals in many social settings, and perhaps especially in a mate-choice context, will involve the perception of fragrance and body odor blends, rather than either the fragrance or body odor alone. This, coupled with the findings from the current study and those of Lenochová et al. (2012), highlights the potential importance of the fragrance choice decision that individuals make. It has been shown, for example, that fragrance preferences are linked to idiosyncratic genetic traits such as MHC (Milinski and Wedekind, 2001), but future research should focus on elucidating the fragrance choice process that individuals undergo, assessing the relative role of genetics but also other factors such as commercial advertising, which are likely to be influential in this process.

Clearly more work is needed to further elucidate the effects of fragrance on individual discrimination, as well as understanding the process related to fragrance choice, but the current study has provided some ground work which will be useful for directing future research in this area. The main findings are in keeping with previous literature discussed, supporting the idea that individual fragrance choice does not mask information present in body odor, though further research is needed to clarify the difference between odor discrimination of male and female odors. Finally, while we have found evidence to suggest that personal fragrance choice does not prevent the overall discrimination of an individual, further investigation must be carried out to ascertain whether fragrance use masks other kinds of information that may be available in body odor, such as emotions, health status and fertility status.

## Author Contributions

CA and SR designed the study. CA collected the data and wrote the manuscript. CA, SR, and JH were all involved in analysis and interpretation of the data, as well as revising the manuscript.

### Conflict of Interest Statement

The authors declare that the research was conducted in the absence of any commercial or financial relationships that could be construed as a potential conflict of interest.
